# Metabolic Phenotype and Adipose Tissue Inflammation in Patients with Chronic Obstructive Pulmonary Disease

**DOI:** 10.1155/2010/173498

**Published:** 2010-12-21

**Authors:** Peter Skyba, Jozef Ukropec, Pavol Pobeha, Barbara Ukropcova, Pavol Joppa, Timea Kurdiova, Katarina Stroffekova, Miroslav Brusik, Iwar Klimes, Ivan Tkac, Daniela Gasperikova, Ruzena Tkacova

**Affiliations:** ^1^Department of Respiratory Medicine, Faculty of Medicine, P.J. Safarik University and L. Pasteur Teaching Hospital, 04001 Kosice, Slovakia; ^2^Institute of Experimental Endocrinology, Slovak Academy of Sciences, 83306 Bratislava, Slovakia; ^3^Department of Biophysics, Faculty of Natural Sciences, P.J. Safarik University, 04001 Kosice, Slovakia

## Abstract

Potential links between metabolic derangements and adipose tissue (AT) inflammation in patients with chronic obstructive pulmonary disease (COPD) are unexplored. We investigated AT expressions of interleukin (IL)-6, tumor necrosis factor (TNF)-*α*, CD68 (macrophage cell surface receptor), caspase-3, and Bax, and their relationships to the metabolic phenotype in nine cachectic, 12 normal-weight, 12 overweight, and 11 obese patients with COPD (age 62.3 ± 7.2 years). With increasing body mass index, increases in AT expressions of IL-6, TNF-*α*, and CD68 were observed (*P* < .001; *P* = .005; *P* < .001, resp.), in association with reduced insulin sensitivity (*P* < .001). No differences were observed between cachectic and normal-weight patients in AT expressions of inflammatory or proapoptotic markers. Adipose tissue CD68 and TNF-*α* expressions predicted insulin sensitivity independently of known confounders (*P* = .005; *P* = .025; *R* ^2^ = 0.840). Our results suggest that AT inflammation in obese COPD patients relates to insulin resistance. Cachectic patients remain insulin sensitive, with no AT upregulation of inflammatory or proapoptotic markers.

## 1. Introduction

Low-grade systemic inflammation is considered a hallmark of COPD and a mechanism potentially linking COPD to increased rate of systemic manifestations of this disease. Both cachexia and obesity with/without the metabolic syndrome (MetS) represent two poles of metabolic abnormalities that are prevalent in patients with COPD and are related to adverse clinical outcomes [1]. Multiple studies demonstrated relationships between systemic inflammation, reflected by increased inflammatory mediators in the systemic circulation and metabolic derangements in COPD patients [2–8]. Nevertheless, to our best knowledge no data were published on adipose tissue inflammation in either obese or cachectic patients with this disease. This contrasts with a wealth of studies that have demonstrated close links between inflammatory processes within the adipose tissue, metabolic impairment, and either obesity [9, 10] or cachexia [11, 12] in disorders other than COPD.

 White adipose tissue is currently viewed as a highly dynamic endocrine organ that, apart from its metabolic function, releases a wide variety of products with both systemic and local effects [13, 14]. The infiltration of adipose tissue with macrophages aggravates local production of inflammatory cytokines such as interleukin (IL)-6 and tumor necrosis factor-alpha (TNF-*α*) that, in turn, initiate a negative set of events on adipocyte function including impaired lipolysis and reduced insulin responsiveness [15]. The production of IL-6 and TNF-*α* is increased in adipose tissue of obese individuals [9, 10], and adipose tissue inflammation is considered to represent an important pathogenetic factor in the development of obesity-related complications such as insulin resistance [16]. Of note, in patients with COPD, increases in IL-6 and TNF-*α* in the systemic circulation have been linked to obesity [2] and insulin resistance [3]; nevertheless, no reports analyzed the role of inflammation within the adipose tissue itself in metabolic derangements in such patients. 

 Among patients with COPD and normal body mass index (BMI), approximately 10% suffer from selective wasting of fat-free mass (FFM) [17, 18]. Nevertheless, in most cachectic patients with advanced COPD, parallel loss of both muscle and fat mass occurs [5, 19]. Although several studies suggested that there is a link between COPD-related wasting and systemic inflammation [6, 20], mechanisms and effects of this inflammatory response are not clear. Studies in adipocyte cell lines and in animal models suggest that inflammatory cytokines such as TNF-*α* may reduce the size of adipose tissue depots by several mechanisms, including acceleration of apoptosis [9]. Protein encoded by tumor necrosis factor receptor superfamily 1A (TNFRSF1A) gene is one of the major receptors in TNF-*α* signaling pathway that activates nuclear factor kappa-B, mediates apoptosis, and acts as a regulator of inflammation [21]. Therefore, a question arises whether COPD-related cachexia is associated with local adipose tissue inflammation, and, if so, whether adipose tissue inflammation relates to the activation of proapoptotic processes with subsequent loss of fat mass. 

Adipose tissue expressions of proinflammatory and proapoptotic markers, and their potential links to metabolic derangements among either cachectic or obese patients with COPD are unexplored. Therefore, in the present study we investigated expressions of proinflammatory IL-6, TNF-*α*, TNFRSF1A, CD68 (macrophage cell surface receptor), and of proapoptotic caspase-3 (CASP3) and Bax, and their relationships to the metabolic phenotype in patients with COPD.

## 2. Methods

### 2.1. Subjects

Patients with diagnosis of COPD according to the American Thoracic Society/European Respiratory Society guidelines [22], free from exacerbation for **≥**8 weeks, were recruited from two out-patient clinics affiliated with the university hospital setting. The MetS was diagnosed according to the recent International Diabetes Federation definition [23]. Patients with BMI **<** 20.0 kg·m^−2^ and fat-free mass index **<** 17.0 kg·m^−2^ (males) or **<**14.0 kg·m^−2^ (females) were considered cachectic [24, 25]. Exclusion criteria were use of systemic corticosteroids, treatment with long-term home oxygen therapy, respiratory disorders other than COPD, known diabetes or other metabolic or endocrine disorder, chronic autoimmune, hepatic or renal disease, malignancy, overt cardiac failure, blood coagulation disorder, or therapy with warfarin. Dyspnea severity was evaluated using the Modified Medical Research Council (MMRC) scale [26]. The distance traveled within 6 minutes (6MWD) was recorded without use of supplemental oxygen. Information regarding exacerbations in the preceding year, and medication use was retrieved from patients' charts of the referring physicians. This study is a part of an ongoing study on the metabolic consequences of COPD (MOPD) and had approval of the institutional Ethics Committee. All subjects gave written consent to the study.

### 2.2. Pulmonary Function Tests

Pulmonary function tests were evaluated with the use of bodyplethysmography (Ganshorn, Germany). All pulmonary function testing was performed according to the European Respiratory Society standards with the patients in a sitting position by the same technician in order to ensure consistency of the technique. Three technically acceptable measurements were performed in each patient, and the highest value was included in the analyses. COPD severity was evaluated on the basis of GOLD recommendations [27].

### 2.3. Body Composition

Body composition was obtained using Dual Energy X-Ray Absorptiometry (DEXA) with fan-beam technology (Lunar Prodigy, GE Healthcare). Body weight, fat free mass, and fat mass were adjusted for height squared as the fat free mass and fat mass indices (BMI, FFMI, FMI, resp.).

### 2.4. Insulin Sensitivity

To determine insulin sensitivity, we used a 2-hour hyperinsulinemic-euglycemic clamp with a primed-continuous insulin infusion rate of 1 mIU·min^−1^ per kg body weight and a variable 20% glucose infusion adjusted every 5 min to maintain plasma glucose within 0.5 mmol·L^−1^ (±10%) of target glucose level (5.0 mmol·L^−1^) [28]. The mean amount of glucose infused during the last 40 minutes was used to calculate the rate of whole-body glucose uptake (*M* value). Serum insulin levels (*I*) were measured at 15 min intervals. Insulin sensitivity is expressed as the *M*/*I* ratio averaged over the final 40 min of the clamp. 

### 2.5. Biochemical Analyses

In all patients, peripheral venous blood samples from the antecubital vein were collected between 7.00 and 8.00 AM after 10 hour fast. Serum insulin was determined with electrochemiluminescence immunoassay kits (Elecsys) on Roche Elecsys 1010/2010 and modular analytics E170 immunoassay analyzers (Roche Diagnostics GmbH, Mannheim, Germany); plasma glucose was measured by the glucose oxidase method on a Beckman autoanalyzer. High-sensitivity serum C-reactive protein (CRP) levels were assessed by immunoturbidimetric method (Randox, UK). The analytical sensitivity of this CRP assay is of 0.1 mg/L. Serum TNF-*α* and IL-6 levels were measured using commercially available enzyme-linked immunosorbent assay kits (Beckmann-Coulter Immunotech). Serum free fatty acids (FFA) levels were determined using a colorimetric assay (Randox, UK). At the time of venous blood samples collection, arterial blood sample was obtained by puncture of radial artery for blood gas analysis. 

### 2.6. IL-6, TNF-*α*, TNFRSF1A, CD68, CASP3, and Bax Expressions

Abdominal subcutaneous adipose tissue was taken by aspiration with a bioptic needle (Medin, Czech Republic) under local intracutaneous anesthesia with 1% mesocain after an overnight fast. The samples were quickly washed in saline to eliminate blood and other connective tissue, immediately frozen in liquid nitrogen, and stored at −80°C until analysis. Total RNA was isolated using the RNeasy Lipid Tissue mini kit (Qiagen, Germany); DNAse treatment was included. RNA quantity, purity, and integrity were determined with the microfluidic chips Experion RNA analysis kit (BioRad) as well as with nanophotometer (IMPLEN, Germany). Reverse transcription was performed with aid of High Capacity RNA to cDNA kit (Applied Biosystems, USA). Gene expression was measured in duplicates with aid of the real-time PCR (RotorGene 2000 real-time cycler, Corbett Research, Australia) using the TaqMan Gene Expression Assays (Applied Biosystems, USA). Comparative quantification method (ΔΔCt) was used to calculate the relative gene expression [29].

Adipocyte diameter was assessed histomorphologically on the 5 *μ*m thick sections of adipose tissue stained with Hematoxylin & Eosin. The cell diameter was determined using the ImageJ freeware (UTHSCA, USA). Average diameter of at least 100 cells from each adipose tissue section was calculated.

### 2.7. Statistical Analyses

Statistical analyses were performed using SPSS software version 14.0 (SPSS Inc., USA). Power calculations were performed based on published IL-6 mRNA expressions in human subcutaneous adipose tissue [30]. For standard deviation of 2.6 in four studied groups, a power calculation indicated that we would need a total sample size of at least 38 patients to detect a difference of 2 ΔΔCt in IL-6 mRNA expression with a power of 80% at a 0.05 significance level. 

The Kolmogorov-Smirnov test of normality was applied. Differences between groups in normally distributed variables were tested by analysis of variance (ANOVA) with Bonferroni's *post hoc* test, and in nonnormally distributed variables by ANOVA on ranks with Dunn's *post hoc* test. Fisher exact test was used to compare the proportion of categoric variables between groups. To assess the relationships between selected variables, linear regression analysis was used. Because the distributions of mRNA expressions and serum cytokine concentrations were all skewed, we used the log-transformed values of these variables in regression analyses. In the multivariate analyses, multiple linear regression models were used with insulin resistance (*M*/*I* value) as the dependent variable, and age, gender, forced expiratory volume in 1 second (FEV_1_), BMI, and log transformed adipose tissue mRNA expressions as independent variables. Continuous variables are shown as means ± SD, nonnormally distributed variables as median (interquartile range).

## 3. Results

### 3.1. Patients

Forty-four patients with COPD (38 men and 6 women) were enrolled. They were generally late middle-aged (mean age 62.3 ± 7.2 years) with a mean 33.8 ± 25.9 pack years history of smoking. Patients were divided into four groups: the first was formed by nine cachectic patients (BMI < 20.0 kg·m^−2^), the second by 12 normal-weight (BMI 20.0–24.9 kg·m^−2^), the third by 12 overweight (BMI 25.0–29.9 kg·m^−2^), and the fourth by 11 obese (BMI ≥ 30.0 kg·m^−2^) patients.  Table 1 displays demographic data, body composition parameters, pulmonary functions, and circulatory inflammatory markers in the four studied groups. No differences were observed in the demographic characteristics among the groups. Cachectic patients had significantly lower, whereas obese patients had significantly higher FFMI and FMI compared to normal-weight patients (*P* < .05 for all comparisons). 

With increases in BMI, FEV_1_, and FEV_1_/forced vital capacity (FVC) ratio and diffusion capacity for carbon monoxide significantly increased (*P* = .007, *P* = .019, *P* < .001, resp.), while residual volume (RV), total lung capacity (TLC), and RV/TLC ratio significantly decreased (*P* = .007, *P* = .026, *P* = .029, resp.) (Table 1). No differences were observed between the four groups in serum levels of IL-6, TNF-*α*, and high sensitivity CRP. None of the patients in the cachectic group fulfilled the criteria for the MetS, whereas these criteria were met by 2 of 12 normal-weight, 5 of 12 overweight, and 10 of 11 obese patients.  Table 2 displays parameters of the respective components defining MetS in the studied groups. 

### 3.2. Insulin Sensitivity and Free Fatty Acids

From cachectic to normal-weight, overweight, and obese patients with COPD, there were significant reductions in insulin sensitivity, in association with higher FFA at the steady state of euglycemic hyperinsulinemic clamp (*P* < .001; *P* = .004, resp.) (Table 3).

### 3.3. Adipose Tissue Expression of IL-6, TNF-*α*, TNFRSF1A, and CD68

**Figure 1:**
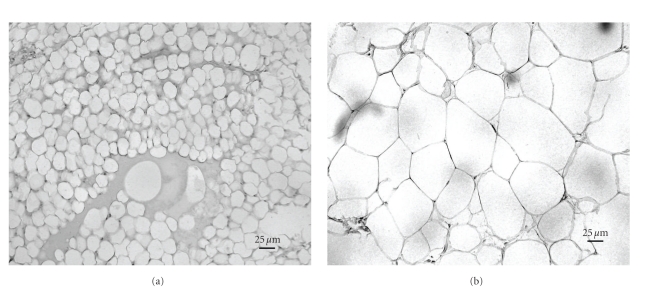
Histomorphological picture of adipose tissue in a cachectic (a) and obese (b) patient with chronic obstructive pulmonary disease (×200 magnification).

 Significant increases in the adipose tissue mRNA expressions of IL-6, TNF-*α*, TNFRSF1A, and CD68 were observed from cachectic to normal-weight, overweight, and obese COPD patients (*ANOVA* for trend, *P* < .001; *P* = .005; *P* = .011, *P* = .011, resp.), in association with increases in adipocyte diameter (*P* < .001) (Table 4). Histomorphological picture of subcutaneous adipose tissue derived from a cachectic and overweight participant in this study is illustrated in Figure 1. Compared to normal-weight patients, obese subjects had significantly higher adipose tissue expressions of CD68 and TNF-*α* expressions. CD68 expression correlated directly with IL-6, TNF-*α*, and TNFRSF1A expressions (*r* = 0.493, *P* < .001; *r* = 0.421, *P* = .005; *r* = 0.509, *P* < .001, resp.). Importantly, no differences were observed between cachectic and normal-weight groups in any of the studied adipose tissue mRNA expressions. Also, no relationships were observed between adipose tissue expressions of IL-6, TNF-*α*, or CD68 and concentrations of inflammatory mediators in the systemic circulation, FEV_1_, or PaO_2._

Adipose tissue expressions of IL-6, TNF-*α*, and CD68 correlated all directly with BMI and adipocyte diameter, and inversely with insulin sensitivity (Table 5). To further strengthen analyses of these data and to eliminate the potential impact of cachectic patients on these relationships, we performed regression analyses in a subgroup of COPD patients after exclusion of those suffering from cachexia. These analyses indicated that even when cachectic patients were excluded, relationships remained significant between insulin sensitivity and CD68, IL-6, and TNF-*α* expressions (*r* = −0.563, *P* < .001; *r* = −0.473; *P* = .004; *r* = −0.459, *P* = .005, resp.). CD68 and TNF-*α* expressions remained significantly associated with BMI (*r* = 0.498, *P* = .002; *r* = 0.438, *P* = .007, resp.), whereas the relationship between IL-6 expression and BMI became nonsignificant (*r* = 0.310, *P* = .066). 

In multiple linear regression analysis with insulin sensitivity (*M*/*I* value) as the dependent variable, and age, gender, BMI, FEV_1_, log CD68, log TNF-*α*, and log IL-6 as independent variables, four variables were independent predictors of insulin sensitivity: age (*P* < .001), BMI (*P* < .001), and log transformed adipose tissue CD68, and TNF-*α* expressions (*P* = .005, *P* = .025, resp.; *R* ^2^ = 0.840).

### 3.4. Adipose Tissue Expression of CASP3 and Bax

Adipose tissue expressions of proapoptotic CASP3 and Bax did not differ among cachectic, normal-weight, overweight, and obese patients with COPD (Table 4), and no relationships between insulin sensitivity and CASP3 or Bax expressions were observed (*r* = 0.162, *P* = .413; *r* = 0.122, *P* = .446, resp.). In addition, inflammatory cytokine expressions were not related to those of either CASP3 or Bax (Table 5).

## 4. Discussion

The present study provides several novel observations on the links between adiposity, adipose tissue inflammation, and insulin resistance in patients with COPD. Our data demonstrate that obesity is linked to increased adipose tissue expressions of CD68 and TNF-*α*, and that both, CD68 and TNF-*α* expressions predict insulin sensitivity independently of known confounders. Cachectic patients were not insulin resistant, and neither upregulation of proinflammatory CD68, TNF-*α*, or IL-6, nor increases in proapoptotic CASP3 or Bax expressions were observed in adipose tissue of such subjects. 

Obesity has emerged as an important risk factor for respiratory disorders [31] that, in association with other components of the MetS, may represent an underlying mechanism contributing to increased cardiovascular risk in COPD. The presence of three or more components of the MetS was demonstrated in almost 50% of COPD patients [32]. In the large-scale epidemiological study in 121,965 participants in France, three factors were inversely related to lung function: atherogenic dyslipidemia (low high-density lipoprotein cholesterol, high triglycerides), “glucose-blood pressure” factor (high fasting glycemia, high blood pressure), and abdominal obesity (large waist circumference) [33]. Other studies also demonstrated high prevalence of arterial hypertension among patients with COPD: Marquis et al. [32] reported raised blood pressure in 82%, and Watz et al. [34] in up to 77% of patients with this disorder. Of note, hypertension was highly prevalent across all GOLD stages of COPD severity, and pulmonary function was not related to increases in blood pressure [34]. In contrast, obesity was strongly associated with higher prevalence of all components of the MetS including hypertension [32]. In agreement, our findings demonstrated significant stepwise increases in the presence of central obesity, arterial hypertension, raised triglycerides, and raised fasting plasma glucose with increasing BMI category. 

Inflammation within the adipose tissue has profound adverse effects on insulin signaling pathways [35]. *In vitro* studies demonstrated that chronic exposure to IL-6 induces insulin resistance in skeletal muscle through induction of JNK, SOCS-3, and protein tyrosine phosphatase 1B [36]. In addition, upregulation of both IL-6 and TNF-*α* impairs biological effects of insulin also in adipose tissue [9, 37]. In normoxemic noncachectic patients with COPD, associations between reduced insulin sensitivity and increased IL-6 concentrations in the systemic circulation have been previously observed [3]. Our study extends this observation further and suggests that adipose tissue inflammation reflected by increases in CD68 and TNF-*α* expressions plays an important role in the whole-body insulin resistance in patients with COPD. 

Macrophages are a significant source of proinflammatory molecules in most tissues [16]. In the present study, we observed relationships between high adiposity, insulin resistance, and the adipose tissue expression of macrophage cell surface receptor CD68. In addition, adipose tissue CD68 expressions were related to the expressions of TNF-*α* and IL-6 suggesting that macrophages might play a central role in the promotion of inflammation within the adipose tissue of COPD patients. Importantly, in contrast to some investigations implying that, within the adipose tissue, adipocytes are both the main source and target of proinflammatory signals, other recent studies indicate that macrophages within the stromal vascular fraction produce most of the proinflammatory molecules [38]. Indeed, Permana et al. [39] demonstrated that TNF-*α* produced by adipose tissue macrophages promotes the appearance of the adipocyte “inflammatory” phenotype and is also related to insulin resistance, thus suggesting that the cross-talk between adipose tissue macrophages and adipocytes generates a vicious circle of aggravating adipose tissue inflammation and insulin resistance [40]. Exploration of the respective contribution of the adipocyte versus stromovascular fraction to the overall adipose tissue inflammatory pattern was beyond the scope of the present study and requires further investigation.

Our results demonstrating increases in adipose tissue inflammation in association with increased trunk fat mass in obese COPD patients raise an intriguing question: might the local cytokine production from adipocytes from/around the lungs contribute to the inflammatory processes within the lungs and potentially to lung function impairment? Interestingly, greater expressions of leptin within the bronchial submucosa were previously observed in COPD patients compared to healthy controls, in association with greater expression of activated T lymphocytes [41]. In addition, a positive association between sputum concentrations of leptin and TNF-*α* in stable patients with COPD suggests a role of adipocytes in regulating airway inflammation in COPD [42]. These investigations coupled by the results of the present study suggest that it is worthwhile to explore the interactions between the local adipose tissue inflammation and bronchial inflammation in the future.

Cachexia has been identified as a significant determinant of mortality in COPD, independent of lung function, smoking, and BMI [42, 43]. In malignant and nonmalignant conditions including COPD, the main mechanisms underlying the loss of muscle mass are a fall in protein synthesis and increase in protein degradation, in association with accelerated muscle cell death [11]. In contrast, little is known regarding mechanisms underlying the loss of adipose tissue mass [44]. Studies in adipocyte cell lines and in animal models demonstrated that tissue inflammation might represent an important contributing mechanism to fat loss. Inflammatory cytokines such as TNF-*α* can reduce fat depot size by several means: by increasing lipolysis and decreasing lipoprotein lipase activity, by decreasing lipogenic enzymes, by down-regulating the adipogenic differentiation factors, and by increasing adipose tissue apoptosis [45]. To this end, we analyzed relationships between the fat cell size and the expressions of inflammatory and proapoptotic molecules in the present study. Contrary to our expectations, adipose tissue expressions of proapoptotic and inflammatory markers were not increased in cachectic patients, and CD68 or TNF-*α* expressions were not related to those of either proapoptotic CASP3 or Bax. Therefore, activation of proapoptotic processes was unlikely to be responsible for adipose tissue loss in our patients. Interestingly, similar observations were reported recently in patients with cancer cachexia. In the report by Warne [46], mRNA expressions of IL-6 and other cytokine and leukocyte markers were not altered in cachectic patients with cancer, and no major fat cell death was present either. Of note, the small size of adipocytes in patients with COPD observed in the present study was similar to that observed in patients with cancer-related cachexia [47]. Future studies are needed to assess the likely role of increased lipolysis and/or reduced lipogenesis in adipose tissue loss. 

There are several limitations to this study. First, our analyses are based on single measurements of insulin sensitivity and the respective adipose tissue mRNA expressions, which may not reflect these relationships over time. Therefore, this study does not provide information about the time-course relationship between metabolic derangements and activation of proinflammatory gene expression in adipose tissue. In addition, factors related to the translation of the mRNA into a stable or active protein have not been studied, and analyses on the relationships between the mRNA expressions of adipokines and their actual protein levels within the adipose tissue remain to be explored in the future. Second, the present study was not designed to address directly the question of the origin of systemic inflammation in patients with COPD. Analysis of the adipose tissue contribution to the overall systemic inflammatory pattern requires measurements of arteriovenous differences in the concentrations of inflammatory mediators [48], an approach used previously by Fontana et al. [49]. Such approach was beyond the scope of the present investigation. Third, the limited number of patients studied in four respective groups is a limitation of this study. However, compared to cachectic and normal-weight COPD patients, those with obesity had median values of adipose tissue expressions of CD68 higher by 240% and 89%, and of TNF-*α* by 88% and 109%, respectively. We therefore believe that our results are robust to gain some understanding of adipose tissue inflammation in cachectic and obese COPD patients. Also, it has to be underlined that concomitant measurements of body composition, insulin resistance, and adipose tissue expressions of inflammatory cytokines within one group of patients with COPD are unique in the pulmonary literature and represent the strength of the study. 

In summary, the present study demonstrated relationships between obesity, adipose tissue inflammation reflected by increased adipose tissue expressions of CD68 and TNF-*α*, and insulin resistance in patients with COPD. Cachectic patients remain insulin sensitive, with no adipose tissue upregulation of inflammatory or proapoptotic markers. Future studies are needed to investigate the role of impaired lipolysis, lipogenesis, and/or aging on adipose tissue structure and function in such patients. From the clinical perspective, the impact of excessive fat mass on the overall cardiovascular risk and prognosis of patients with COPD and possible implications for nonpharmacological and pharmacological interventions need to be explored.

## Figures and Tables

**Table 1:** Patient characteristics.

Variable	Entire cohort	Group				*P* value
		Cachectic	Normal weight	Overweight	Obese	
Patients, no. (%)	44 (100)	9 (20.5)	12 (27.3)	12 (27.3)	11 (25)	.867
Age, yr	62.3 ± 7.2	61.4 ± 8.2	62.8 ± 5.9	64.3 ± 7.0	60.2 ± 8.0	.580
Males, no. (%)	38 (86.3)	9 (100)	10 (83.3)	10 (83.3)	9 (81.8)	.615
Smokers, no. (%)	17 (38.6)	4 (44.4)	7 (58.3)	2 (16.7)	4 (36.4)	.207
Packyears, yr	33.8 ± 25.9	40.7 ± 15.7	38.8 ± 25.7	37.6 ± 35.2	19.2 ± 15.4	.232

MMRC dyspnea scale	2.0 (1.0-2.0)	2.0 (2.0-2.0)	1.0 (1.0-2.0)	2.0 (1.0-2.0)	2.0 (1.0-2.0)	.228
6MWD, m	363.8 ± 89.8	296.1 ± 86.4	388.4 ± 93.0	385.2 ± 73.7	365.2 ± 91.3	.105
BMI, kg·m^−2^	26.5 ± 7.1	18.3 ± 0.8^†^	22.9 ± 1.4	27.7 ± 1.4^∗†^	35.9 ± 5.6^∗†^	<.001
FFMI, kg·m^−2^	18.3 ± 2.5	16.1 ± 0.8	17.4 ± 1.4	18.2 ± 1.5*	21.2 ± 2.6^∗†‡^	<.001
FMI, kg·m^−2^	8.2 ± 5.2	4.7 ± 1.3^†^	6.2 ± 1.4	8.9 ± 1.5*	14.1 ± 5.3^∗†‡^	<.001
Trunk FM, %	30.5 ± 14.3	10.5 ± 7.6	25.0 ± 8.4*	39.6 ± 3.3^∗†^	44.3 ± 5.0^∗†^	<.001
Trunk FM, kg	14.2 (6.3–17.7)	1.3 (1.2–5.1)	9.5 (5.4–10.6)	15.9 (15.5–16.9)*	21.4 (20.7–27.6)^∗†^	<.001
Limbs FM, %	25.0 ± 12.9	11.4 ± 6.2	19.0 ± 6.3	29.8 ± 9.8^∗†^	38.2 ± 9.8^∗†^	<.001
Limbs FM, kg	5.0 (3.2–7.7)	1.4 (0.9–2.6)	3.4 (3.0–3.8)	5.9 (5.5–6.9)*	9.4 (7.7–14.0)^∗†^	<.001

FEV_1_, L	1.58 ± 0.70	1.1 ± 0.9	1.5 ± 0.5	1.9 ± 0.7	1.7 ± 0.6	.084
FEV_1,_ % pred	54.0 ± 22.9	33.8 ± 19.8	51.4 ± 16.8	65.7 ± 25.0*	60.5 ± 19.0*	.007
FEV_1_/FVC ratio	50.5 ± 16.7	38.7 ± 15.2	46.5 ± 13.0	57.0 ± 19.2	58.6 ± 13.2*	.019
RV, L	4.13 ± 1.39	5.3 ± 1.4	4.4 ± 1.1	3.6 ± 1.3*	3.4 ± 1.0*	.007
TLC, L	7.29 ± 1.50	8.1 ± 1.1	7.8 ± 1.4	6.8 ± 1.6	6.5 ± 1.3	.026
RV/TLC ratio	55.6 ± 10.6	64.3 ± 13.7	55.4 ± 7.4	51.4 ± 8.6*	52.5 ± 9.5	.029
DL_CO_, % pred	72.3 ± 30.9	34.2 ± 24.3	67.1 ± 15.1	87.1 ± 37.2*	92.0 ± 7.6*	<.001
PaO_2_, mm Hg	69.0 ± 13.5	58.7 ± 19.5	69.8 ± 8.3	75.2 ± 14.3*	68.9 ± 7.5	.048
PaCO_2_, mm Hg	38.3 ± 6.8	39.8 ± 8.0	35.7 ± 3.9	38.5 ± 7.1	38.9 ± 7.2	.495

Leukocytes, ×10^9^·L^−1^	7.2 ± 1.7	7.1 ± 2.5	7.0 ± 1.7	7.5 ± 1.1	7.2 ± 1.7	.921
Neutrophils, ×10^9^·L^−1^	4.2 ± 1.3	4.4 ± 2.0	4.1 ± 1.5	4.5 ± 0.7	3.9 ± 0.9	.738
IL-6, pg·mL^−1#^	3.7 (2.9–5.5)	4.3 (3.2–5.2)	2.9 (2.0–3.8)	3.8 (3.0–5.8)	4.4 (3.0–8.6)	.352
TNF-*α*, pg·mL^−1#^	13.4 (10.6–17.5)	9.8 (8.6–14.1)	13.1 (11.0–18.1)	13.9 (11.3–20.1)	15.2 (11.3–17.3)	.214
hsCRP, mg·L^−1#^	3.1 (1.0–7.8)	3.5 (0.8–7.4)	1.4 (0.9–7.2)	2.7 (1.1–12.0)	3.6 (1.2–4.6)	.932

*P < .05 versus cachectic group.

^†^ *P* < .05 versus normal-weight group.

^‡^ *P* < .05 versus overweight group.

Values are given as the mean ± SD, unless otherwise indicated.

^#^Values are given as median (interquartile range).

MMRC: The Modified Medical Research Council Dyspnea Scale; 6MWD: 6-minute walking distance; BMI: body mass index; FFMI: fat-free mass index; FMI: fat mass index; FM: fat mass; FEV_1_: forced expiratory volume in one second; FVC: forced vital capacity; RV: residual volume; TLC: total lung capacity; DL_CO_: carbon monoxide diffusing capacity; PaO_2_: arterial oxygen partial pressure; PaCO_2_: arterial carbon dioxide partial pressure; IL-6: interleukin 6; TNF-*α*: tumor necrosis factor alpha; hsCRP: high sensitivity C-reactive protein.

**Table 2:** Characteristics of the respective components of the metabolic syndrome in COPD patients.

	Group				*P* value
	Cachectic	Normal weight	Overweight	Obese	
Central obesity*	0 (0)	6 (50.0)	12 (100)	11 (100)	<.001
Raised blood pressure*	2 (22.2)	4 (12.5)	8 (66.6)	11 (100)	.001
Raised triglycerides*	0 (0)	1 (8.3)	1 (8.3)	7 (63.6)	<.001
Reduced HDL cholesterol*	0 (0)	1 (8.3)	0 (0)	3 (27.3)	.089
Raised fasting plasma glucose*	0 (0)	5 (41.6)	4 (33.3)	8 (72.7)	.010

Values are given as No (%).

*Central obesity: waist circumference > 94 cm (men) or >80 cm (women); raised blood pressure: systolic BP ≥ 130 mm Hg or diastolic BP ≥ 85 mm Hg, or treatment of previously diagnosed hypertension; raised triglycerides: >150 mg·dL^−1^ or specific treatment; reduced HDL cholesterol < 40 mg·dL^−1^ (men) or <50 mg·dL^−1^ (women), or specific treatment; raised fasting plasma glucose > 100 mg·dL^−1^ or specific treatment.

HDL: high density lipoprotein; BMI: body mass index.

**Table 3:** Insulin sensitivity determined by euglycemic hyperinsulinemic clamp and insulin-induced serum-free fatty acids in patients with COPD.

Variable	Entire group	Group				*P* value
		Cachectic	Normal weight	Overweight	Obese	
*M*, mg·kg^−1^·min^−1^	5.00 ± 2.74	7.48 ± 2.22	6.42 ± 2.65	3.81 ± 1.84^∗†^	2.71 ± 1.20^∗†^	<.001
*M*/*I* × 100, mg·kg^−1^·min^−1^·*μ*U^−1^·mL	6.72 ± 4.50	11.65 ± 3.52	9.01 ± 3.65	4.76 ± 2.62^∗†^	2.33 ± 1.01^∗†‡^	<.001
FFA, mmol·L^−1^	0.11 ± 0.05	0.07 ± 0.03	0.09 ± 0.04	0.12 ± 0.06	0.14 ± 0.05^∗†^	.004

*P < .05 versus cachectic group.

^†^ *P* < .05 versus normal-weight group.

^‡^ *P* < .05 versus overweight group.

*M*: glucose uptake; *M/ I*: insulin sensitivity—glucose uptake to insulinemia ratio; FFA: free fatty acids.

**Table 4:** Adipose tissue-relative expressions of interleukin 6, tumor necrosis factor-alpha and its receptor, CD68, CASP3, and Bax gene in patients with COPD.

mRNA expression (ΔΔCt)	Entire group	Group				*P* value
		Cachectic	Normal weight	Overweight	Obese	
IL-6	3.1 (1.3–4.3)	0.9 (0.5–1.0)	2.4 (1.4–4.7)	3.8 (2.9–4.8)*	3.9 (3.1–4.9)*	<.001
TNF-*α*	5.5 (4.2–7.1)	5.1 (3.4–6.0)	4.6 (3.3–6.0)	5.5 (5.2–6.4)	9.6 (6.1–10.2)^∗†‡^	.005
TNFRSF1A	9.7 (8.0–11.8)	8.1 (6.4–9.5)	9.7 (8.2–11.9)	9.8 (8.1–11.7)	11.3 (9.7–15.3)	.011
CD68	9.9 (7.9–16.7)	5.3 (4.6–8.1)	9.5 (6.7–11.0)	11.7 (9.3–17.2)*	18.0 (11.2–23.5)^∗†^	.011
CASP3	5.3 (4.7–6.7)	5.3 (4.9–6.0)	4.7 (3.9–7.8)	5.0 (4.4–6.9)	6.2 (5.7–6.7)	.890
Bax	2.2 (0.4–8.6)	0.9 (0.3–2.8)	3.3 (0.6–10.1)	1.4 (0.4–4.6)	6.5 (0.3–9.5)	.175
Adipocyte diameter (*μ*m)	68.2 (59.0–77.0)	48.1 (37.7–63.0)	60.6 (57.7–65.2)	69.7 (65.9–78.7)*	77.0 (75.3–77.9)^∗†^	<.001

*P < .05 versus cachectic group.

^†^ *P* < .05 versus normal-weight group.

^‡^ *P* < .05 versus overweight group.

Values are given as median (interquartile range).

Relative mRNA expression was calculated using comparative quantification method (ΔΔCt).

IL-6: interleukin 6; TNF-*α*: tumor necrosis factor alpha; TNFRSF1A: tumor necrosis factor superfamily receptor 1A; CASP3: Caspase 3.

**Table 5:** Linear relationships between IL-6, TNF-*α*, and CD68 relative expressions in adipose tissue and insulin sensitivity, BMI, adipocyte diameter, FEV_1_, arterial oxygen saturation, and serum inflammatory markers.

	IL-6 (Log ΔΔCt)		TNF-*α* (Log ΔΔCt)		CD68 (Log ΔΔCt)	
	*R* value	*P *value	*R* value	*P *value	*R* value	*P *value
*M/I, *mg·kg^−1^·min^−1^·*μ*U^−1^·mL	−0.716	<.001	−0.438	.003	−0.425	.004
BMI, kg·m^−2^	0.565	<.001	0.448	.003	0.445	.003
Adipocyte diameter, *μ*m	0.593	<.001	0.347	.024	0.469	.002
FEV_1_, % pred	0.245	.108	0.055	.727	0.014	.929
PaO_2_, mm Hg	0.224	.145	0.023	.882	0.015	.924
CASP3 mRNA expression, (ΔΔCt)	0.249	.107	0.172	.259	0.023	.902
Bax mRNA expression, (ΔΔCt)	0.232	.142	0.094	.560	0.120	.454
Serum TNF-*α* (Log pg·mL^−1^)	−0.234	.131	−0.013	.934	0.178	.253
Serum IL-6 (Log pg·mL^−1^)	0.232	.139	0.271	.083	0.364	.018
hsCRP (Log mg·L^-1#^)	0.180	.247	0.220	.157	0.212	.171

PaO_2_: arterial oxygen partial pressure; *M/I*: insulin sensitivity—glucose uptake to insulinemia ratio; BMI: body mass index; FEV_1_: forced expiratory volume in 1 second; CASP3: caspase 3; TNF-*α*: tumor necrosis factor alpha; IL-6: interleukin-6; hsCRP: high sensitivity C-reactive protein.

## Abbreviations

BMI: Body mass index
CASP3: Caspase-3
COPD: Chronic obstructive pulmonary disease
CRP: C-reactive protein
DEXA: Dual Energy X-ray Absorptiometry
FEV_1_: Forced expiratory volume in one second
FFA: Free fatty acids
FFM: Fat free mass
FFMI: Fat free mass index
FMI: Fat mass index
FVC: Forced vital capacity
GOLD: Global Initiative for Chronic Obstructive Lung Disease
IL: Interleukin
MetS: Metabolic syndrome
MMRC: Modified Medical Research Council
PaCO_2_: Arterial carbon dioxide partial pressure
PaO_2_: Arterial oxygen partial pressure
RV: Residual volume
SD: Standard deviation
TLC: Total lung capacity
TNF-*α*: Tumor necrosis factor-*α*
TNFRSF1A: Tumor necrosis factor receptor superfamily 1A
6MWD: Six minutes walk distance.
